# Morphological, physiological, and biochemical responses of three different soybean (*Glycine max* L.) varieties under salinity stress conditions

**DOI:** 10.3389/fpls.2024.1440445

**Published:** 2024-09-09

**Authors:** Desilal Kokebie, Abiyu Enyew, Getinet Masresha, Tarekegn Fentie, Emebet Mulat

**Affiliations:** ^1^ Department of Biology, College of Natural and Computational Sciences, University of Gondar, Gondar, Ethiopia; ^2^ Department of Chemistry, College of Natural and Computational Sciences, University of Gondar, Gondar, Ethiopia

**Keywords:** *Glycine max*, antioxidant, agronomic traits, proline, salinity, ROS

## Abstract

Salinity is one of the most detrimental factors for the growth performance and productivity of crops worldwide. Therefore, understanding crop responses or growth potentials and their effectiveness in salinity mitigation is highly important for the selection of salinity-tolerant plant varieties. In this study, the effects of salinity at various stress levels (0 mM, 50 mM, 100 mM, and 150 mM NaCl) on the morphological, physiological, and biochemical parameters of three soybean varieties (‘Afigat’, ‘Gishama’, and ‘Pawi-2’) were investigated. The results showed that salinity significantly reduced morphological traits including plant height, number of leaves per plant, stem thickness, shoot and root length, and fresh and dry weight. This reduction was more prominent in the ‘Afigat’ variety for all of these traits except shoot and root length. The concentrations of chlorophyll *a* and *b* decreased with increasing salinity. In addition, salinity significantly increased leaf electrolyte leakage (EL), lipid peroxidation, proline accumulation, and phenol and flavonoid content. The ‘Pawi-2’ variety was more tolerant than the other studied varieties in terms of membrane stability (less EL and a low malondialdehyde content) and proline, phenol, and flavonoid accumulation. Therefore, ‘Pawi-2’ may be considered as the most salt-tolerant variety in comparison with the other studied soybean varieties. Further complementary studies in field conditions including anatomical parameters are needed to confirm these findings.

## 1 Introduction

Salinity is one of the most detrimental environmental stressors and drastically reduces crop growth performance and productivity worldwide (Khan et al., 2021; Ji et al., 2022; Foliar et al., 2023). The salinization of land is a severe ecological issue worldwide, steadily increasing by approximately 10% each year (Joshi et al., 2023). It may negatively affect up to 50% of arable land by 2050 (Sabra et al., 2012; Ke and Hou, 2021). Thus, salinity threatens the agricultural sector and food security in many countries of the world, including Ethiopia (Qureshi et al., 2018; Irin and Hasanuzzaman, 2024). It is expected to increase in the next decade globally (Khan et al., 2021; Ricardo et al., 2023). It is a significant obstacle to the growth of agriculture and hinders sustainable development in Ethiopia (Adhanom, 2019; Ahmed et al., 2019). Approximately 44 million hectares, or 36% of the country’s total land area, are potentially susceptible to salinity issues. Of these, 11 million hectares are in the Rift Valley and other arid and semi-arid lowland regions, which make up more than half of the country’s total land area (Qureshi et al., 2018). Exacerbating factors for land salinization include constantly shifting weather patterns, high evapotranspiration rates, extensive fertilizer application, poor irrigation practices, inadequate on-farm water management, and poor drainage systems (Aredehey et al., 2018). Owing to this, a sizable portion of once arable land in the country has been officially taken out from agricultural production due to rising salinity (Tessema et al., 2023).

Salinity stress affects plant growth and productivity by inducing osmotic stress, ionic stress, or a combination of them (Dichala and Giannakoula, 2022). Following salinity stress, an excessive build-up of sodium (Na^+^) and chloride (Cl^-^) ions results in ionic toxicity and the production of reactive oxygen species (ROS) within plant cells (Hasanuzzaman et al., 2022). Salinity leads to oxidative damage primarily by the production of excess ROS, which can target proteins, lipids, and DNA (Zhou et al., 2018; Hasanuzzaman et al., 2022). ROS are oxygen derivatives created by diverse cellular metabolic pathways in distinct cellular compartments (Hasanuzzaman et al., 2022) and are byproducts of metabolic activities (Singh, 2022). ROS, such as hydrogen peroxide (H_2_O_2_), superoxide (O_2_), singlet oxygen (^1^O_2_), and hydroxyl radical (OH^-^), can be generated under normal growth conditions. However, abiotic stressors such as salinity induce the overproduction of ROS, which in turn damages the cellular function of plants (Kao, 2017; Kesawat et al., 2023). ROS-induced oxidative stress injures the cell membrane, reduces plant biomass production, impairs osmotic adjustment and the electron transport chain in chloroplasts and mitochondria, inhibits protein synthesis, and limits water and nutrient uptake. Plants have developed different coping mechanisms for mitigating ROS-induced oxidative damage through osmotic adjustment via the accumulation of compatible solutes, such as proline (Ahmed et al., 2019), and the enhancement of antioxidant defense systems, such as flavonoids and phenolic compounds (Hanifah and Purwestri, 2021; Ricardo et al., 2023), as an adaptation mechanism to abiotic stressors, including salinity. The biosynthesis of metabolites such as flavonoids and phenolic compounds plays a vital role in protecting plants from stress by scavenging or detoxifying ROS and increasing plant tolerance against stress (Zhou et al., 2018; Hasanuzzaman et al., 2021).

Soybean (*Glycine max* L.) is one of the most nutritionally and commercially important lowland cash crops and belongs to the Fabaceae family. It is a food and feed crop that is used extensively in Ethiopia for various purposes, including human consumption, animal feed, fish and poultry meal, and cash revenue, and is an excellent intercrop (Alebel et al., 2019). Soy-based foods are healthy, high in nutrients, and provide an excellent supply of vital minerals such as potassium, sodium, magnesium, sulfur, phosphorus, and calcium (Mussema et al., 2022). Furthermore, it serves as a fundamental component in the production of foods such as bread, porridge, chapatti, yogurt, soy milk, protein, and ‘shero wot’ in Ethiopia (Ejeta, 2020) and is the most important component of human food, animal feed, green bioenergy, and edible oil production worldwide (Hasanuzzaman et al., 2016; Mussema et al., 2022). It is known as “the meat that is grown on plant” because of its economic importance and high-quality protein supplementation (Anwar-ul-haq et al., 2020) and is known as the ‘golden bean’ or ‘protein hope of the future’ due to its excellent nutritional value (Trina et al., 2021). Approximately 18% of the country’s oilseed production comprises soybean crops (Molla et al., 2024). All these roles have made soybean crops popular in Ethiopia. However, its production is not in line with its demand due to various biotic and abiotic stress factors. Among the factors that diminish yield and productivity, salinity is one of the most common detrimental abiotic factors (Argaw, 2014; Anwar-ul-haq et al., 2016; Hasanuzzaman et al., 2016; Alharby et al., 2021).

Previously, several initiatives have been taken to mitigate the possible adverse effects of salinity stress on soybean plant production, including genetic improvement of varieties via plant breeding (Hunde Desissa, 2019), the use of growth-promoting microbial isolates or bioinoculants (Argaw, 2014; Ertump et al., 2019; Abulfaraj and Jalal, 2021), and other exogenous foliar applications (Rahmawati and Damanik, 2018). However, these methods are not always feasible or environmentally friendly, and some are costly and may cause additional adverse effects, including the re-enhancement of salinity (Khan et al., 2021). In addition, previous studies (Hunde Desissa, 2019; Awoke and Alem, 2021) also reported that identification of salt-tolerant and environmentally adaptive soybean varieties is required. There is also little information available related to the salt tolerance mechanisms of soybean varieties. Hence, we studied the morphological, physiological, and biochemical responses of soybean varieties under salinity stress conditions, and our current study aimed to identify salt-tolerant soybean varieties and understand how they can attenuate salinity-induced oxidative stress via osmotic adjustment and antioxidant defense systems.

## 2 Materials and methods

### 2.1 Soil sample selection and preparation

Soil and cow dung manure were collected from nearby agricultural areas of the University of Gondar, Ethiopia, and the husks and other unnecessary detritus were removed. Determining the physicochemical properties of soil is crucial for understanding how water and nutrients travel through plants and soil profiles (Abeje et al., 2021). Accordingly, the physicochemical parameters of the soil were examined prior to sowing the different soybean varieties (**Table 1**). With a few modifications, the hydrometer methods of Mwendwa (2020) were used to determine the particle size distribution of the soil samples. The soil particle size distribution was determined by using the United States Department of Agriculture (USDA) textural triangle (Pradeep et al., 2021). A digital pH meter (LMPH-10, India) was used to determine the acidity or alkalinity of the soil sample. The electrical conductivity was measured using a digital conductivity meter (Model 601, India). The soil composite was then transferred into the same-sized plastic pots with a top diameter of 25 cm, bottom diameter of 18 cm, and height of 26 cm.

**Table 1:** Physicochemical properties of the experimental soil.

	Particle distribution	Unit	Method
Physical properties	Clay	30%	Hydrometer
	Sand	32%	
	Silt	38%	
	Soil textural class	Clay loam	
Chemical properties	Moisture content	7.72%	Gravimeter
	Electrical conductivity	0.42 mSm^-1^	Conductivity meter
	pH value	7.27	pH meter

### 2.2 Plant materials and experimental design

Seeds of three soybean varieties (‘Afigat’, ‘Gishama’, and ‘Pawi-2’) were obtained from the Gondar Agricultural Research Center (GARC), Ethiopia (**Table 2**). The seeds were subjected to 5 min of surface sterilization using a 5% (w/v) commercial bleach sodium hypochlorite solution (NaOCl), followed by multiple rinses with distilled water and overnight drying. The experiment was conducted at the Botanical Laboratory, Department of Biology, University of Gondar, Ethiopia. The experimental setup was arranged in a randomized complete block design (RCBD) with three replicates. The pots (3 varieties, 4 salt treatments, and 3 replicates, yielding 36 experimental units or pots) were placed in a semi-controlled environment.

**Table 2:** List of soybean varieties selected in this study.

Type of variety	Code of variety	Year of release/register	95% maturity period	Name of breeder/maintainer
‘Afigat’	TGX-1892-10F	2007	121 days	AWARC/SRARI
‘Gishama’	PR-143(-26)	2010	97 days	PARC
‘Pawi-2’	PARC-2013-3	2015	110 days	PaweARC/EIAR

Source: Ministry of Agriculture Variety Registration Books since 2007–2017.

### 2.3 Salt treatment and growth conditions

Six uniform and healthy seeds of each variety were sown per pot with soil and cow dung at a 2:1 ratio and irrigated with tap water every 2 days, and the mean day temperature was approximately 25 ± 3°C. Two weeks after sowing, the seedlings were thinned to four seedlings per pot with a similar growth pattern and comparable vigor. 21 days after sowing, plants were exposed to salt treatment with various concentrations (0 mM, 50 mM, 100 mM, and 150 mM NaCl) (Hasanuzzaman et al., 2022), and each pot received 500ml of such saline solutions in every 2 days-interval. The control treatment (0 mM NaCl) received the same quantity of tap water. The salt treatment was continued for up to 45 days. After 45 days of salt treatment, the plant samples were harvested, and different morphological, physiological, and biochemical parameters were measured.

### 2.4 Measurement of morphological traits

Measurements were made for morphological traits, such as the number of leaves per plant, plant height, stem thickness, and length and weight of the shoots and roots. As recommended by Fatema et al. (2023), the total number of leaves per plant per pot was counted, and the average was used to determine the number of leaves per plant for every treatment. The plant height of each variety was measured from the base to the top or the apical bud of the plants using a ruler and reported in centimeters. Stem thickness was measured using a digital handy caliper meter (CM145, UK), and the unit of measurement was reported in millimeters. At the end of the experiment, plant samples of each variety were collected, and the shoot and root parts were separated for measurement using scissors. The shoot length was measured from the shoot-root junction to the tip of the longest branched leaf, whereas the root length was measured from the shoot-root junction to the tip of the tape root using a ruler. The fresh weight (FW) of the shoots and roots was immediately measured by weighing them on a digital electrical balance (CY510, Citizen Scale, Poland), and their dry weight (DW) was measured after drying in a hot air oven at 70°C for 72 h.

### 2.5 Measurement of physiological traits

#### 2.5.1 Chlorophyll pigments determination

Photosynthetic pigments such as the chlorophyll (chll *a* and chll *b*) contents of the leaves were determined using the method described by Arnon (1949). One gram of fresh leaves was weighed and extracted with 20 ml of 80% acetone. After centrifugation, the absorbance (A) was measured at 645 nm and 663 nm using a UV-VIS spectrophotometer. Finally, the concentrations of chlorophyll pigments (i.e., chll *a* and chll *b*) were calculated using the following formula (the unit is µg g^-1^ FW).


$${\text{Chlorophyll a (mgg}}^{-1}\text{FW)}=\left[12.7\left({\text{A}}_{663nm}\right)-2 .69\left({\text{A}}_{645nm}\right)\times \frac{V}{1000W}\right]$$

$${\text{Chlorophyll b (mgg}}^{-1}\text{ FW)}=\left[22.9\left({\text{A}}_{645nm}\right)-4.68\left({\text{A}}_{663nm}\right)\times \frac{V}{1000W}\right]\;$$

where A is the absorbance, V is the total volume of the filtrate, and W is the leaf FW.

#### 2.5.2 Electrolyte leakage

With slight modifications, the method of Dionisio-Sese and Tobita (1998) was used to estimate the electrolyte leakage (EL) of leaves, a marker of the degree of membrane damage. Leaf disks were made by punching three randomly selected leaf samples from each pot using a 1 cm stainless steel cork borer. These leaf disks (two disks for each leaf) were submerged immediately in a test tube filled with 10 ml of distilled water and shaken for 5 h. After 5 h of incubation, the initial electrical conductivity (EC1) was measured using a digital conductivity meter (Model 601, India). The solution containing the leaf disks was again immersed in a water bath heated at 100°C for 30 min, followed by cooling in an ice box for 10 min. Finally, the EL of the leaf leachates was ascertained by measuring the electrical conductivity of this solution (EC2):


$$\text{Electrolye leakage (EL\%)}=\frac{{\text{EC}}_{1}}{{\text{EC}}_{2}}\times 100$$

### 2.6 Measurement of biochemical traits

#### 2.6.1 Membrane lipid peroxidation (MDA content)

Malondialdehyde (MDA), the decomposition product of the polyunsaturated fatty acid (PUFA) component of the membrane under stress conditions, was measured to detect membrane lipid peroxidation. With slight modifications, the thiobarbituric acid (TBA) method of Heath and Packer (1968) was used to determine the level of MDA, the end product of lipid peroxidation. Three fresh leaf samples were obtained from each plant, cut finely using scissors and dried in a hot air oven at 60°C for 24 h. Then, 0.5 g of dried leaf composites were extracted with 10 ml of 0.1% (w/v) trichloroacetic acid (TCA) solution using a mortar and pestle. The mixture was then centrifuged at 8,000 rpm for 30 min, and 1 ml of the supernatant was mixed with 4 ml of 20% TCA containing 0.5% TBA. The combined solution was then heated to 100°C in a water bath for 30 min, followed by cooling in an ice bath for 5 min. Finally, using 20% TCA with 0.5% TBA as a blank, the specific and non-specific absorbance were measured at 532 nm and 600 nm, respectively. The MDA content was calculated by using Lambert’s extinction coefficient, ϵM= 155 mM^-1^ cm^-1^, and expressed as nmol g-1 DW.


$${\text{MDA equivalents (nanomoleg}}^{-1}\text{ DW})=\frac{1000}{155}\left({\text{A}}_{532\text{nm}}-{\text{A}}_{600}\text{nm}\right)\times \frac{\text{dilution factor}}{\text{gram of sample}}$$

#### 2.6.2 Determination of leaf proline content

The acid-ninhydrin method proposed by Bates et al. (1973) was used to determine the proline content of leaves using D-proline as a standard. From each replicate, approximately 250 mg of dried leaf composite was extracted with 5 ml of 3% 5-sulfosalicylic acid using a mortar and pestle, and the mixture was centrifuged for 30 min at 8,000 rpm. After adding 1 ml of acid ninhydrin solution and 1 ml of glacial acetic acid to 1 ml of supernatant, the mixture was incubated for 1 h at 100°C in a water bath. Finally, the mixed solution was extracted using 2 ml of toluene by vortexing for 5 min, and the absorbance was read at 520 nm using toluene as a blank. The proline content was determined from the standard curve equation (*Y* = 9.6154𝑥 −16.667; *R* ^2 ^= 0.9947) (**Supplementary Figure A**), and its unit was expressed as µg g*^−^* ^1^ DW.

#### 2.6.3 Determination of total phenol content

The Folin–Ciocalteu (FC) technique was used to measure the total phenol content (TPC). Using a digital analytical precision balance (Model JA203H, India), 250 mg of dried leaf composite was measured and extracted with 10 ml of distilled water. Then, 1 ml of the extracts was transferred to a 10 ml volumetric flask containing 5 ml of distilled water and 1 ml of FC reagent. The mixture was left to stand at room temperature for 10 min. One milliliter of 7.5% Na_2_CO_3_ was then added to this reaction mixture and re-incubated for 1 h. Finally, a mixture of 7.5% Na_2_CO_3_ and diluted FC reagent was used as a blank, and the absorbance of this blue complex was read at 750 nm. The TPC was estimated from the gallic acid calibration curve (R^2 ^= 0.999), and its unit was expressed as mg GAE/g DW (**Supplementary Figure B**).

#### 2.6.4 Determination of total flavonoid content

The aluminum chloride colorimetric method reported by Chang et al. (2002) with minor modifications was used to determine the flavonoid content of leaves using quercetin as a standard. Ten milliliters of 70% ethanol was used to extract 0.3 g of the dried leaf samples. One milliliter of the extract was transferred to a 10 ml volumetric flask containing 300 µl of 10% AlCl_3_ and 300 µl of 5% NaNO_2_ mixed solution. Then, 1 M of NaOH solution was added to the reaction mixture after 6 min of incubation, followed by re-incubation for 30 min at room temperature. Finally, the absorbance of this yellow-brownish complex solution was read at 510 nm using a UV-visible spectrophotometer. The total flavonoid content was determined from the standard curve of quercetin (**Supplementary Figure C**), and its unit is reported in mgg^-1^DW.

### 2.7 Data analysis

The entire experiment was carried out with three replicates in an RCBD. The data were analyzed using R software version 4.3.2, and the ggplot2 package was used to plot the bar graph. The data are presented as the means ± standard deviations. The statistically significant differences between the means of several groups were compared using Fisher’s least significant difference (LSD) test at the 0.05 significance level.

## 3 Results

### 3.1 Physical and chemical properties of the soil samples

The physicochemical properties of the experimental soil were evaluated before the experiment (**Table 1**). Determining the physicochemical characteristics of the soil was of paramount importance for the growth of plants. Accordingly, the particle size composition of the experimental soil was 32% sand, 38% silt, and 30% clay (**Table 1**), and its textural class was clay loam, which is ideal for soybean plant growth. In addition, the soil sample’s pH was 7.27, which indicates that the soil is somewhat alkaline. According to the USDA soil salinity classification, the soil was categorized as non-saline soil (ECe < 4dSm^-1^). Therefore, the experimental soil used in the pot experiment was considered to be non-saline soil; hence, it had no effect on the growth of the soybean plants.

### 3.2 Morphological, physiological, and biochemical parameter analysis

The findings of this study indicated that significant distinctions across varieties were noted (*p* < 0.05) in all of the studied morphological parameters except the leaf count (**Table 3**) and physio-biochemical parameters except the chlorophyll *a*/*b* ratio (**Table 4**). Salt treatment also significantly affected all the studied parameters (**Tables 3**, **4**). Morphological features, such as shoot dry weight (SDW) and root fresh weight (RFW), and all physio-biochemical parameters, except the TPC and chll *a*: chll *b*, were substantially influenced by the variety-salt treatment interaction. This demonstrated that the degree of variation among varieties was adequate for choosing a salt-tolerant variety to withstand salinity stress. The variety type and salinity stress both had an impact on the majority of the morphological and physio-biochemical parameters, as shown by the considerable differences in the variety, treatment, and variety-salt treatment interaction variances.

**Table 3:** Combined analysis of variance for morphological parameters of soybean varieties under salinity stress.

Source of variation	d.f.	NL	PH	ST	SL	RL	SFW	SDW	RFW	RDW
Variety	2	18.25^ns^	275.86^*^	3.87^*^	215.4^*^	55.11^*^	311.38^*^	120.2^*^	22.39^*^	0.59^*^
Treatment	3	60.59^*^	1005.9^*^	8.39^*^	1112^*^	45.68^*^	3587.8^*^	519.9^*^	22.62^*^	6.38^*^
V_*_T interaction	6	1.59^ns^	38.57^ns^	0.36^ns^	28.46	9.59^ns^	90.64^ns^	22.85^*^	3.73^*^	0.03^ns^
Error	24	1.59	16.8	0.33	18.9	4.26	63.29	4.95	0.75	0.14
CV (%)		5.98	11.7	7.49	27.1	25.3	11.06	13.1	9.07	4.69
Total	35									

NL, number of leaves; PH, plant height; ST, stem thickness; SL, shoot length; RL, root length; SFW, shoot fresh weight; SDW, shoot dry weight; RFW, root fresh weight; RDW, root dry weight; V, variety; T, salt treatment. ^ns^ and ^*^ indicate that the mean difference was not significant and significant at the 5% significance level, respectively, according to Fisher’s LSD test.

**Table 4:** The combined analysis of variance for physio-biochemical parameters of soybean varieties under salinity stress.

Source of variation	d.f.	EL	Chll *a*	Chll *b*	Chll *a*:Chll *b*	Pro	MDA	TPC	TFC
Variety	2	1624.9^*^	3170^*^	13567^*^	29.1^ns^	0.39^*^	20.49^*^	21.18^*^	2402.2^*^
Treatment	3	4117.3^*^	5347^*^	16211^*^	11.2^ns^	2.13^*^	36.38^*^	23.38^*^	3038.7^*^
V_*_T interaction	6	67.46^*^	342^*^	842^*^	0.41^ns^	0.19^*^	2.31^*^	1.98^ns^	170.6^*^
Error	24	11.39	11.8	9.52	0.032	0.009	0.23	1.95	2.55
CV (%)	35	10.31	13.7	11.9	0.197	3.91	11.17	19.3	7.11
Total									

EL, electrical leakage; Chll a, chlorophyll a; Chll b, chlorophyll b; Pro, proline; MDA, malondialdehyde; TPC, total phenol content; TFC, total flavonoid content; V, variety; T, salt treatment. ^ns^ and ^*^ indicate that the mean difference was not significant and significant at the 5% significance level, respectively, according to Fisher’s LSD test.

### 3.3 Effects of salinity on morphological parameters

**Figure 1:**
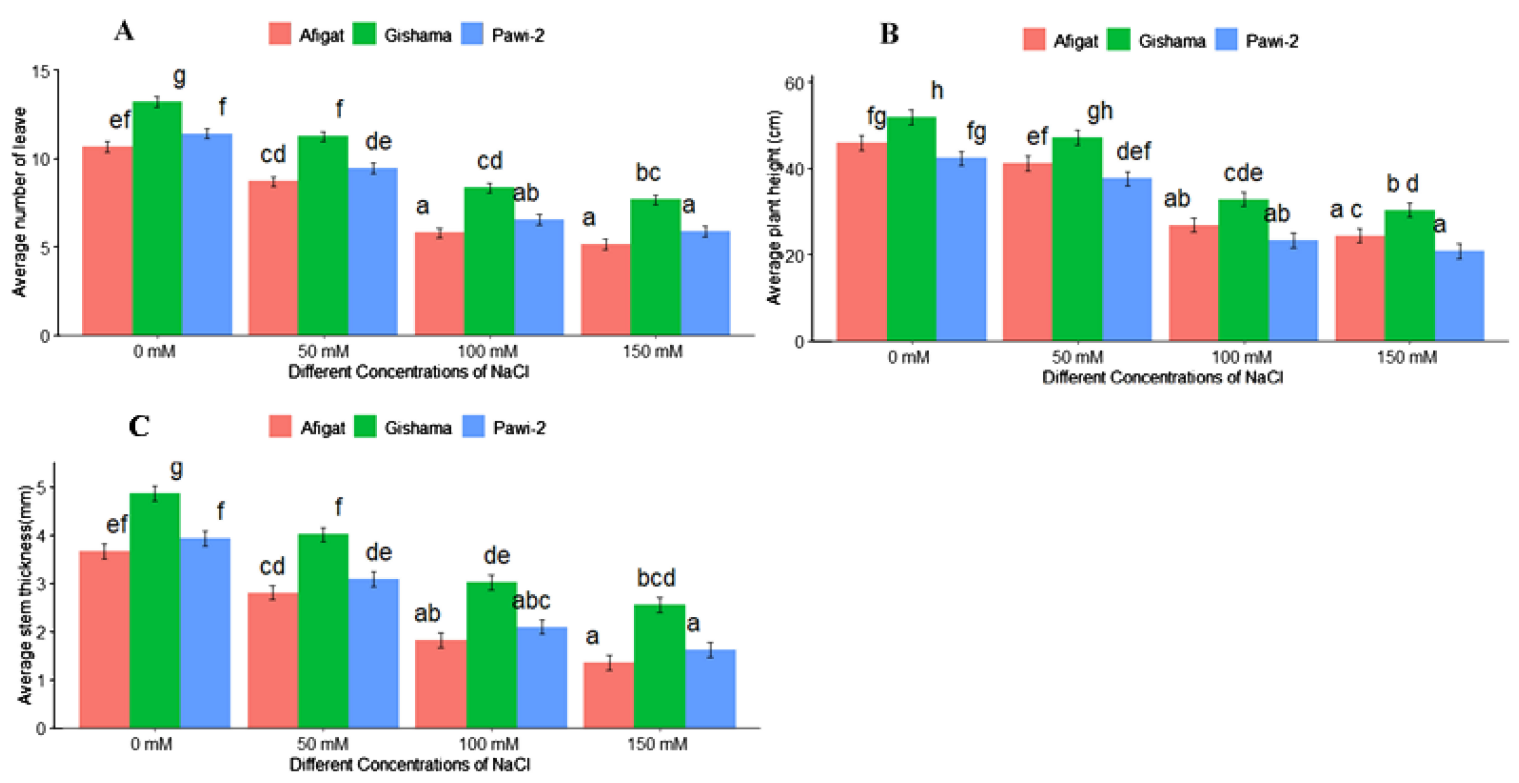
Effects of salinity on the number of leaves **(A)**, plant height **(B)**, and stem thickness **(C)** of three soybean plant varieties. The bars represent the means ± SDs of three replicates. Similar lowercase letters above the vertical bars indicate no statistically significant difference at the 5% significance level according to Fisher’s LSD test.

**Figure 2:**
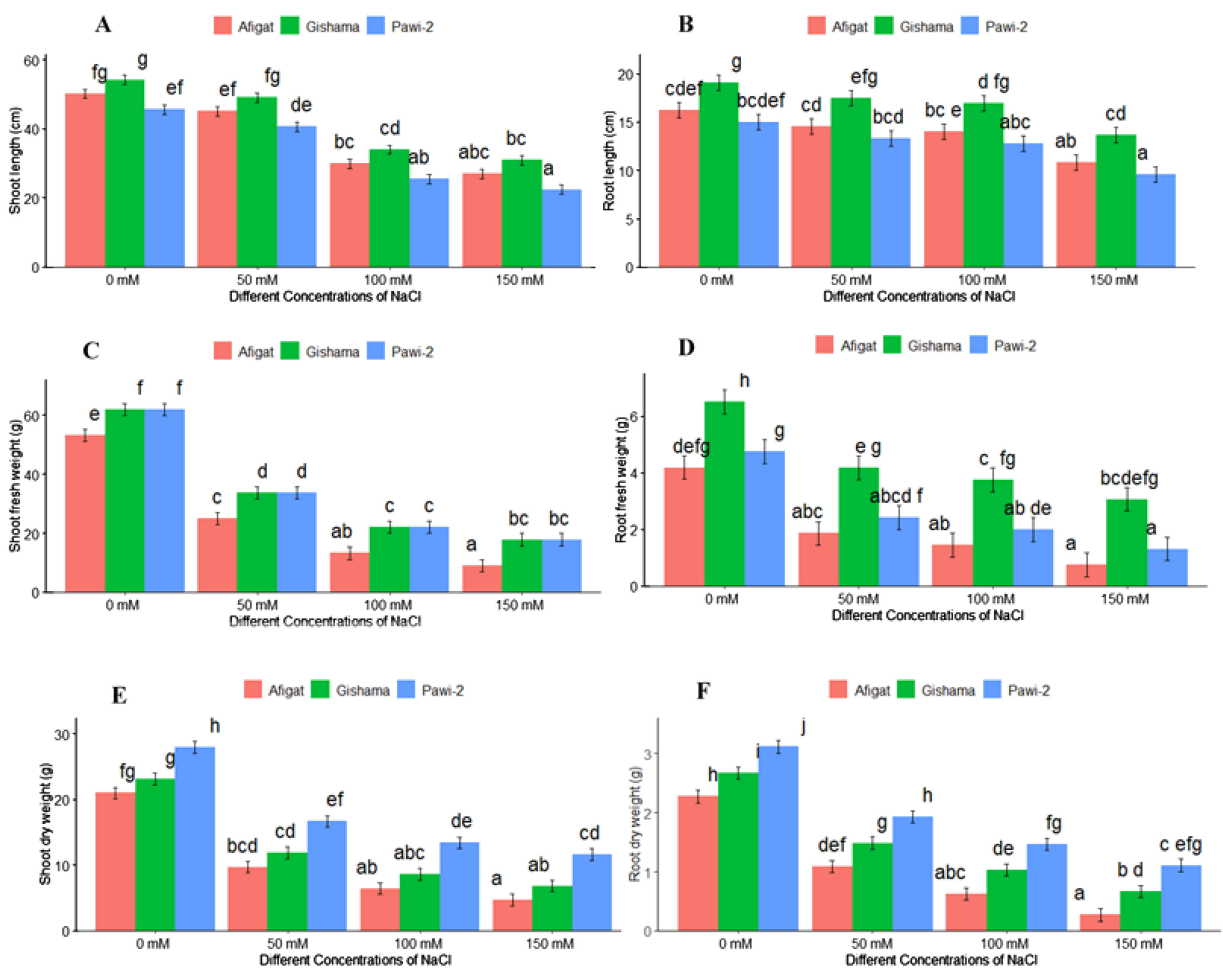
Effects of salinity on shoot length **(A)**, root length **(B)**, shoot fresh weight **(C)**, root fresh weight **(D)**, shoot dry weight **(E)**, and root dry weight **(F)** of three soybean plant varieties. The bars represent the means ± SDs of three replicates. Similar lowercase letters above the vertical bars indicate no statistically significant difference at the 5% significance level according to Fisher’s LSD test.

The effects of salinity stress with various stress labels on the morphological traits of soybean plants are shown in **Figures 1** and **2**. The results revealed that salinity stress sharply decreased morphological traits, such as the number of leaves, plant height, stem thickness, length [shoot length (SL) and root length (RL)], and biomass (FW and DW) of the shoots and roots of all soybean varieties. There was a statistically significant (*p* < 0.05) difference among varieties and salinity labels for all of these traits. However, noticeable differences were not detected between ‘Afigat’ and ‘Pawi-2’ in terms of the number of leaves, plant height, stem thickness, and shoot and root length. In comparison with that of the control, the shoot length of ‘Afigat’ decreased by 8%, 36%, and 49% at 50 mM, 100 mM, and 150 mM NaCl, respectively, and its root length decreased by 4.6%, 27.6%, and 47.9%, respectively, under similar salinity conditions (**Figures 2A, B**). A decrease in SL and RL was more pronounced in the ‘Pawi-2’ variety. Furthermore, an increase in salt concentration had a significant effect on shoot and root fresh weight (**Figures 2C, D**), with no discrete variation between ‘Afigat’ and ‘Pawi-2’ in terms of RFW at any of the salinity labels. Surprisingly, ‘Gishama’ and ‘Pawi-2’ exhibited similar changes in shoot fresh weight in response to salinity stress (**Figure 2C**). Similarly, salinity also markedly reduced the dry mass production (DW) of shoots and roots (**Figures 2E, F**). The effects of salinity on shoot and root biomass production were less pronounced in ‘Pawi-2’. The shoot and root (FW and DW) weights of all the varieties decreased significantly (*p* < 0.05) with increasing salinity stress, and a more drastic effect was visible in the ‘Afigat’ plots. As a result, ‘Pawi-2’ could maintain greater shoot and root biomass production than the other varieties under salt stress conditions. Overall, based on the morphological results, ‘Afigat’ was strongly affected by salinity stress, whereas ‘Gishama’ showed relatively better growth performance under salt stress conditions. Hence, ‘Afigat’ and ‘Gishama’ were identified as the most salt-sensitive and salt-tolerant varieties, respectively.

### 3.4 Effects of salinity on physiological parameters

#### 3.4.1 Chlorophyll pigments

**Figure 3:**
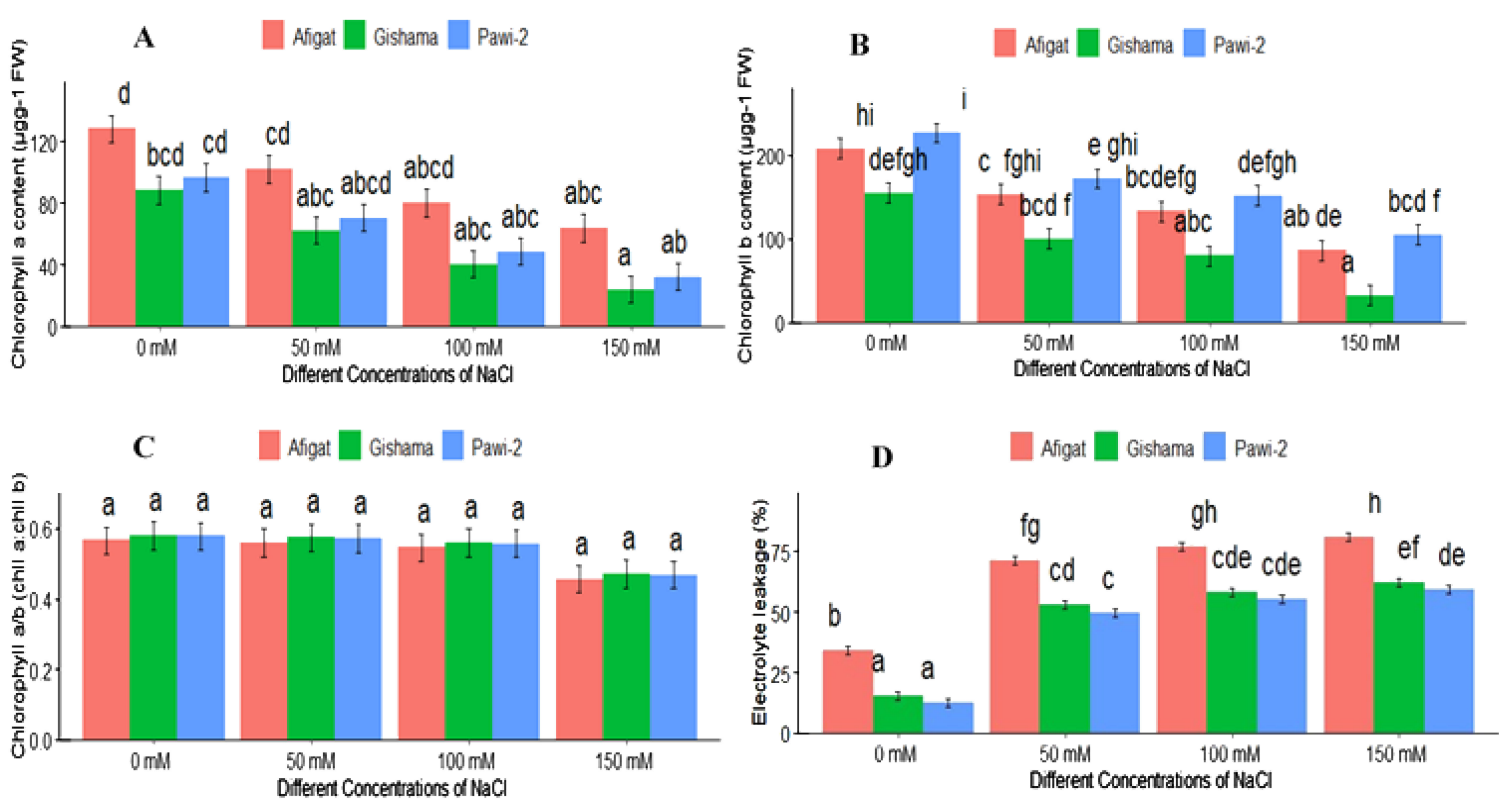
Effects of salinity on chlorophyll *a* content **(A)**, chlorophyll *b* content **(B)**, the chlorophyll *a*/*b* ratio **(C)**, and electrolyte leakage **(D)** of three soybean plant varieties. The bars represent the means ± SDs of three replicates. Similar lowercase letters above the vertical bars indicate no statistically significant difference at the 5% significance level according to Fisher’s LSD test.

The concentrations of chlorophyll pigments (chll *a* and chll *b*) in soybean leaves were strongly affected by salinity stress (**Figure 3**). Chlorophyll *a* and *b* significantly decreased with increasing salinity compared with the control, and statistically significant differences were observed between the control and salt-treated plants. The minimum and maximum reductions in the chll *a* and chll *b* contents were observed in the control and plants treated with the highest salt concentration (150 mM). In ‘Afigat’, the chlorophyll *a* content decreased by 48.4%, 54.5%, and 64%, and the chlorophyll *b* content decreased by 31%, 24%, and 54.5% under 50 mM, 100 mM, and 150 mM NaCl, respectively, compared with the control plants. Moreover, the chlorophyll *a*/chlorophyll *b* ratio (chll *a*:chll *b*) decreased with increasing salinity. However, no discernible difference between treatments or between varieties was observed for the chlorophyll *a*/chlorophyll *b* ratio (**Figure 3C**).

#### 3.4.2 Leaf electrolyte leakage

Compared with the control treatment, the EL in the leaves significantly increased with increasing salinity (**Figure 3D**). Compared with the control, the EL increased by 189%, 242%, and 251% in ‘Afigat’ and by 155%, 181%, and 189% in ‘Gishama’ at 50 mM, 100 mM, and 150 mM NaCl, respectively. Substantial variations were observed among plant varieties in terms of EL. The EL in ‘Afigat’ was greater than that in the other varieties under both the control and salt stress conditions. Furthermore, the EL of "Afigat" was almost two thirds larger than that of "Gishama" and "Pawi-2" all salt concentrations (**Figure 3D**). Nonetheless, significant variations were not detected between ‘Gishama’ and ‘Pawi-2’ in terms of EL, and relatively lower EL was observed in ‘Pawi-2’ than in ‘Gishama’. Hence, ‘Pawi-2’ is more salt tolerant than the other varieties.

### 3.5 The effects of salinity on biochemical parameters

**Figure 4:**
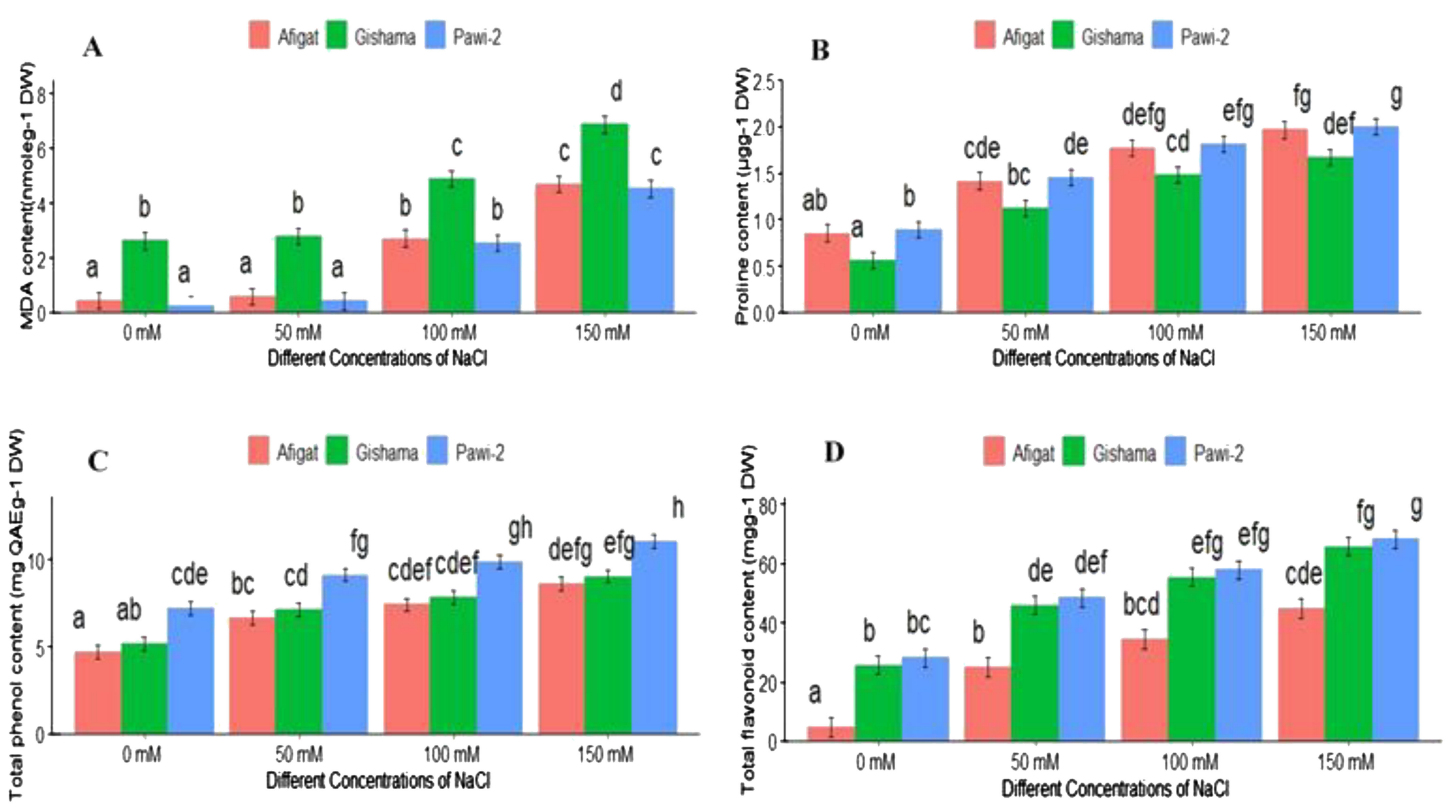
The effects of salinity on the lipid peroxidation-MDA content **(A)**, leaf proline content **(B)**, total phenol content **(C)**, and total flavonoid content **(D)** of three soybean plant varieties. The bars represent the means ± SDs of three replicates. Similar lowercase letters above the vertical bars indicate no statistically significant difference at the 5% significance level according to Fisher’s LSD test.

This study assessed how well different soybean varieties perform against salinity stress in terms of proline accumulation, MDA levels, and total phenol and flavonoid contents. Increasing salinity significantly (p < 0.05) increased the proline, MDA, and total phenol and flavonoid contents, and the highest values were recorded in plants irrigated with the highest salt concentration (**Figure 4**).

#### 3.5.1 Membrane lipid peroxidation (MDA content)

MDA is the end product of the PUFA peroxidation of the membrane under stress conditions. By measuring the amount of MDA, an indicator of lipid peroxidation and membrane damage, the degree of membrane lipid peroxidation under stress conditions was ascertained. **Figure 4A** shows the impact of salinity on the membrane lipid peroxidation of the three soybean varieties. The results showed that the MDA content in all soybean varieties increased with increasing salinity. Compared with the control treatment, the MDA content in the plants treated with 150 mM NaCl was greater, and the other salt concentrations were greater for all the soybean varieties. Substantial variations were observed among the salinity concentrations. However, there was no discernible difference in the MDA content between the control and 50 mM NaCl treatments. The data analysis revealed significant variations (*p* < 0.05) in the MDA content among the varieties. ‘Gishama’ had the highest MDA content (higher lipid peroxidation), and ‘Pawi-2’ had the lowest MDA content (less lipid peroxidation) across all salt concentrations.

#### 3.5.2 Proline content

**Figure 4B** shows the proline content of leaves in response to various NaCl concentrations. The results showed that increasing salinity stress caused a significant increase in the leaf proline content compared with that of the control. The treatment with the highest salinity had the highest proline content, and the control treatment had the lowest proline concentration. The proline contents of ‘Afigat’, ‘Gishama’, and ‘Pawi-2’ were approximately 1.6, 2, and 2.4 times greater, respectively, than those of the corresponding controls at 100 mM NaCl. A notable difference (*p* < 0.05) was observed among the varieties, with the minimum proline accumulation observed in ‘Gishama’. Although there was no significant difference between ‘Afigat’ and ‘Pawi-2’, comparatively greater proline accumulation was detected in the ‘Pawi-2’ variety.

#### 3.5.3 Total phenol content

With increasing salt concentrations, the TPC of the leaves increased compared with that in the control treatment. As shown in **Figure 4C**, the lowest and highest TPCs were observed in the control plants and plants treated with 150 mM NaCl, respectively. Compared with those of the control, the TPCs of ‘Afigat’, ‘Gishama’, and ‘Pawi-2’ increased by approximately 32%, 31.2%, and 40%, respectively, at 100 mM NaCl. Statistically significant differences were observed between the examined varieties. The results of the analysis of variance revealed that there were significant differences (*p* < 0.05) in the TPCs of the leaves across the varieties but not between ‘Afigat’ and ‘Gishama’. The lowest and highest phenolic values were recorded in ‘Afigat’ and ‘Pawi-2’, respectively, across all NaCl concentrations.

#### 3.5.4 Total flavonoid content

**Figure 4D** illustrates the evaluation of the impact of different NaCl concentrations on the total flavonoid content of soybean plant leaves. The results demonstrated that when exposed to salinity, all varieties showed a considerable increase in flavonoid content, and significant differences (*p* < 0.05) were noted among the varieties. The plants treated with the highest dose of salt (150 mM) and the untreated (control) plants had the highest and lowest flavonoid levels, respectively. Compared with the control treatment, the flavonoid content in ‘Pawi-2’ increased by 2.3, 2.8, and 3.4 times, and in ‘Gishima’ increased by 1.9, 2.5, and 2.8 times under 50, 100, and 150 mM NaCl, respectively.

### 3.6 Correlation analysis among traits

**Figure 5:**
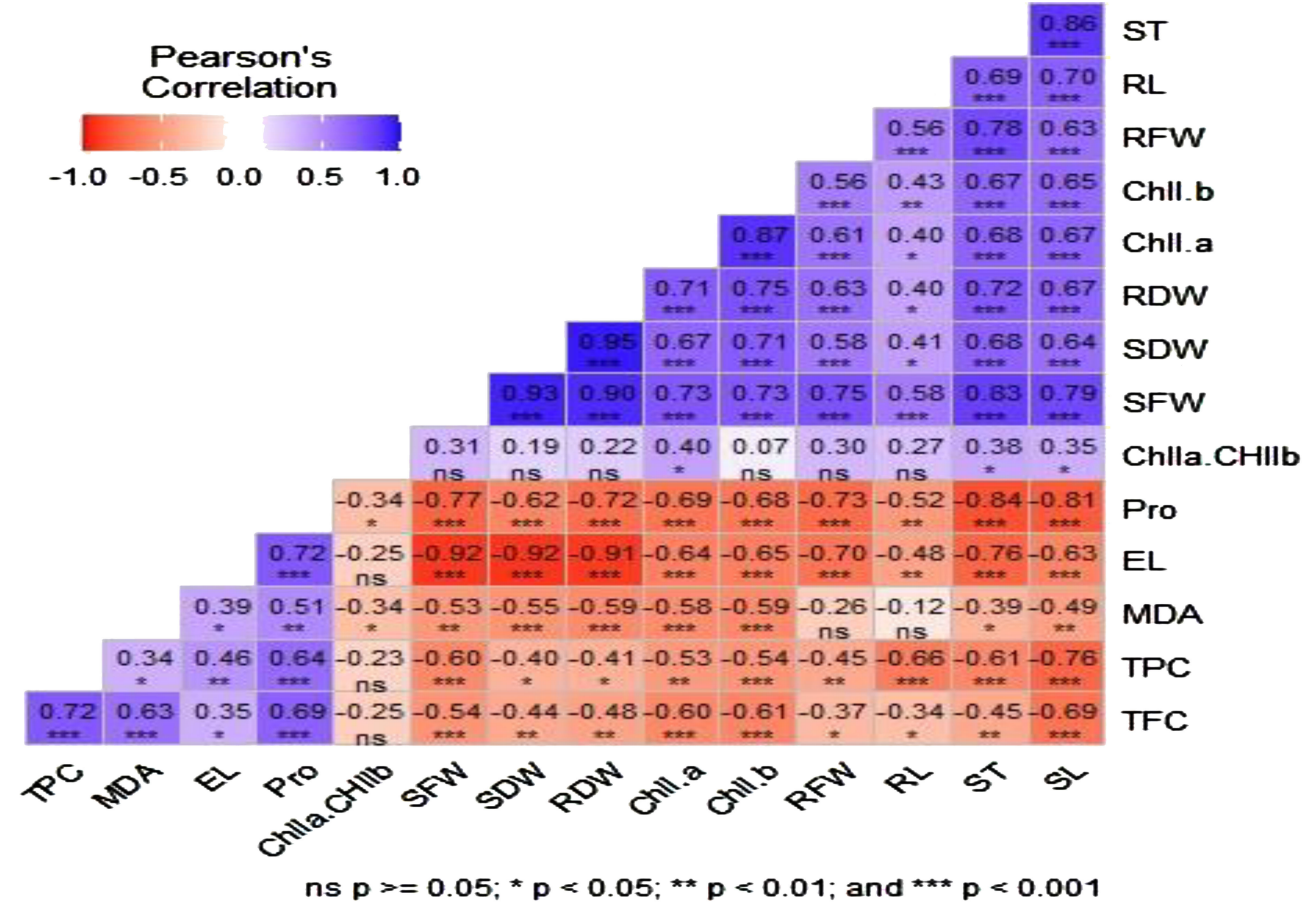
Pearson correlation matrix between different morphological, physiological, and biochemical traits of three soybean varieties under salinity stress conditions. EL, electrolyte leakage; MDA, malondialdehyde; Pro, proline; TPC, total phenol content; TFC, total flavonoid content; Chll *a*, chlorophyll *a*; Chll *b*, chlorophyll *b*; SFW, shoot fresh weight; SDW, shoot dry weight; RFW, root fresh weight; RDW, root dry weight; SL, shoot length; RL, root length; ST, stem thickness. *^ns^*, *, **, and *** indicate no significant difference and a significant difference at *p* < 0.05, *p* < 0.01, and *p* < 0.001, respectively.

Pearson correlation for morphological, physiological, and biochemical traits was performed using the “Metan package” of R software (**Figure 5**). Some measured parameters had a strong negative association, whereas others displayed a positive and significant correlation. For instance, there was a substantial positive relationship between chlorophyll pigments (chll *a* and chll *b*) and the length (SL and RL) and weight (FW and DW) of shoots and roots. Additionally, there was a strong positive correlation between proline accumulation in leaves and EL. However, proline accumulation, EL, and total phenol and flavonoid contents were negatively and significantly associated with shoot and root length. A strong and inverse relationship was also observed between the total phenol concentration and morphological traits such as shoot and root length, stem thickness, and shoot fresh weight.

## 4 Discussion

In the present study, significant differences in the morphological, physiological, and biochemical features of soybean plants were detected, except for the number of leaves. Variety and salt treatment interactions were also found to be significant for morphological traits such as SDW and RFW and for all physio-biochemical traits except the TPC of leaves. Our study findings suggest that the growth performance and biomass production of soybean plant varieties were reduced when salinity stress increased, compared with the control. Similar findings have been reported previously for agricultural crops such as sorghum (Saberi et al., 2011), soybean (Amirjani, 2010), mung bean (Mankar et al., 2021), and lettuce (Foliar et al., 2023). Salinity stress induces osmotic, ionic, and water stresses, which in turn affect plant growth (number of leaves, plant height, stem thickness, SL, and RL), cause defoliation, and stop the growth of new leaves (Mankar et al., 2021; Wasif et al., 2023). These are in harmony with our findings, as we found a significant (*p* < 0.05) reduction in these growth parameters with increasing salinity. Similarly, previous studies (Aini and Setiawan, 2014; Siddiki et al., 2020; Ullah et al., 2020) reported a reduction in plant height, stem thickness, and the number of leaves with increasing salt concentrations. A decrease in growth parameters is associated with the ability of salinity to suppress plant growth through ion toxicity and nutritional imbalances or a combination of these factors (Wasif et al., 2023). In addition, according to earlier research, which corroborated our findings, SL, RL, FW, and DW (both in shoot and root) decreased under salt stress conditions, and the reductions in all these parameters were more prominent at the highest salt concentration (Oprica and Marius, 2014; Anwar-ul-haq et al., 2016; Ahmed et al., 2019; Rahman et al., 2020; Siddiki et al., 2020; Trina et al., 2021; Das et al., 2022). The reduction in SL and RL with increasing salinity may be due to the ionic toxicity effect of salt stress, which inhibits cell elongation and plant growth, stimulating hormone production, such as cytokinesis.

By studying the effect of salt stress on chlorophyll content, the results showed the photosynthetic efficiency of plants in response to salt stress conditions. **Figure 4** shows the significant effect of various concentrations of sodium chloride on chlorophyll content, and an inverse relationship was observed between salt concentration and chlorophyll content. The obtained results agree with those of Heidari (2011), who reported a significant decrease in the chlorophyll content (chll *a* and chll *b*) of basil (*Ocimum basilicum* L.) genotypes with increasing salt concentrations. Similarly, recent studies were also conducted by Hasanuzzaman et al. (2022) and Sonone et al. (2023), who demonstrated that salinity stress results in a decrease in the chlorophyll content (chll *a* and chll *b*) of soybean and rice genotypes. However, some earlier findings contradicted ours, indicating that the concentration of chlorophyll increased with increasing salt stress (Abdul Qados, 2011; Liao and Xu, 2021).

The ratio of chlorophyll *a* to chlorophyll *b* (chll *a*:chll *b*) has an impact on plant photosynthetic efficiency. Its variation plays a significant role in understanding the photosynthetic efficiency and the biosynthesis and degradation pathway of chlorophyll pigments (Nguyen et al., 2021). Sonobe et al. (2020) also revealed a positive correlation between chll *a*:chll *b* and the quantum efficiency of photosystem II. In the present study, the chlorophyll *a*/*b* ratio decreased with increasing salt concentration, which is in agreement with the results reported by Gomes et al. (2017) regarding *Salvinia auriculata* and those of Hasanuzzaman et al. (2022) regarding soybean plants. This is because salinity had a greater and more pronounced effect on chlorophyll *a* than on chlorophyll *b*, making chlorophyll *a* more susceptible to salinity (Rout et al., 1997). This indicates the destruction of the photosynthetic pigments due to salinity stress. However, this result is consistent with Pacheco-Sangerman et al. (2024), who revealed that the ratio of chlorophyll *a* to chlorophyll *b* increased with increasing salinity, with a slight reduction under severe salinity conditions. In addition, Hasanuzzaman et al. (2021) reported higher chlorophyll *a*/*b* in salt-treated plants than in untreated plants. This results in an increased chlorophyll *a/b* ratio due to the increased susceptibility of chlorophyll b to salinity and the reduced production of chlorophyll pigment by the enzyme chlorophyllase (Mohabbati, 2005; Ashraf and Harris, 2013). In addition, the increase in the ratio of chlorophyll *a*/*b* occurs because of the conversion of chlorophyll *b* to chlorophyll *a* as a result of the degradation of the first step of chlorophyll *b* under salt stress conditions (Mane et al., 2010).

Estimating oxidative parameters (EL and MDA) in response to stress, such as salinity, helps in understanding the extent of membrane damage and loss of membrane integrity. In the present study, the contents of oxidative parameters (EL and MDA) significantly increased with increasing salinity, and higher values of these traits were observed at the highest NaCl concentration. The increasing level of EL in salt-stressed plants may be attributed to the triggering effect of salinity on membrane damage. Leaf EL was more prominent in ‘Afigat’. This indicates that salinity-induced oxidative stress caused more significant membrane damage in ‘Afigat’ than in the other plants. This could be due to the poor development of defensive mechanisms in these plants, which are prone to saline environments and the high leaching of ions from leaf tissue. In support of these findings, Hniličková et al. (2019) in lettuce and spinach, Mankar et al. (2021) in mung bean, Ke and Hou (2021) in water drop wort, Rasel et al. (2021) in rice, and Dichala and Giannakoula (2022) in *Punica granatum* L. reported an increase in the EL and MDA content with increasing salt concentrations. The lowest EL and MDA contents were detected in ‘Pawi-2’ compared with the other varieties at all salt concentrations. This indicated that ‘Pawi-2’ is more salt tolerant than others, which is supported by earlier reports from Momeni et al. (2021) and Saxena and Purty (2018), who reported that salt-tolerant plants have lower EL and MDA contents than salt-susceptible plants. A relatively low EL is an indicator of membrane stability and has been linked to salinity tolerance, as reported by Sabra et al. (2012) in three *Echinacea* species. Similarly, in line with our results, the findings of Mankar et al. (2021) in mung bean and Habibi et al. (2021) in tomato reported that the contents of EL and MDA in salt-sensitive plants were greater than those in salt-tolerant plants. It has also been reported that greater EL and MDA accumulation are prominent indicators of plant membrane impairment under salt stress (Khan et al., 2009; Ashraf and Ali, 2010; Carloni et al., 2012; Tuwair et al., 2015; Mankar et al., 2021), as the cell membrane is one of the major primary sites of ROS and causes oxidative damage in plants (Sharma et al., 2012; Dayem et al., 2017; Mankar et al., 2021). The significant positive correlation between EL and MDA content (r = 0.4, *p* < 0.05) (**Figure 5**) suggested that both EL and MDA were indicators of solute leakage and were most likely responsible for the disruption of membrane integrity. Overall, the relatively high tolerance of ‘Pawi-2’ to salinity stress could be associated with improved defensive mechanisms via osmotic adjustment (high proline accumulation) and increased antioxidant activity (increased phenol and flavonoid contents).

During osmotic adjustment, the basic adaptive response of plant cells to salinity is essential for their survival and growth under salt stress conditions. High concentrations of compatible osmolytes such as proline can accumulate in plants exposed to salinity stress. In the current study, we observed that, compared with the control treatment, salinity significantly (*p* < 0.05) increased the proline content in all the soybean varieties. In agreement with our findings, previous studies revealed that the proline content increased with increasing salinity, and a promising increase was detected at the highest salt concentration (Wu et al., 2014; Ajithkumar, 2017; Siddiki et al., 2020; Ke and Hou, 2021). The increase in proline content coupled with increasing salt concentrations has also been reported in many agricultural crops, such as alfalfa (Xiao-shan and Jian-guo, 2009), lettuce (Ahmed et al., 2019), soybean (Das et al., 2022), and wheat (Wasif et al., 2023). Furthermore, an increase in proline has been reported in *Portulaca oleracea* (Kafi and Rahimi, 2011) and *Ocimum basilicum* (Heidari, 2012) under salinity stress conditions. The greater accumulation of proline under stress is due to the increased activity of enzymes involved in proline biosynthesis, the decrease in proline degradation, and the inhibition of proline-catabolizing enzymes (El kholy et al., 2021). According to our results, ‘Pawi-2’ exhibited relatively high proline accumulation among the varieties, whereas ‘Gishama’ showed relatively low proline accumulation at all NaCl concentrations. Therefore, ‘Pawi-2’ is more salt tolerant than the other varieties. This finding is consistent with the findings of Liu and van Staden (2000) and Ke and Hou (2021), who reported that salt-tolerant plants accumulate more proline than salt-sensitive plants. Therefore, the findings of this study confirmed that proline is necessarily produced for osmotic adjustment in plants under salinity stress conditions in response to salinity-induced oxidative stress.

In the present study, we found a noticeable increase in flavonoid and phenolic compound contents with increasing salinity stress, which is in strong agreement with previous studies (Kafi et al., 2011; Kiani et al., 2021) that reported high total phenol and flavonoid contents under increasing salt concentrations. This confirms that secondary metabolites such as flavonoids and phenolic compounds are possibly produced as a defensive mechanism when plants are exposed to stress such as salinity (Sabra et al., 2012). Numerous plants have also been observed to have higher total phenol and flavonoid concentrations under salinity stress than control plants (Chutipaijit et al., 2009; Valifard et al., 2014; Olfa et al., 2018; Zhou et al., 2018; Kiani et al., 2021; Hossain et al., 2022). This was confirmed in our study, as we observed an increase in phenol and flavonoid contents with increasing salt concentrations in all soybean varieties. In the present study, we found a significant positive correlation between antioxidant compounds (phenols and flavonoids) and MDA, with values of r=0.34 (*p* < 0.05) and r= 0.68 (*p* < 0.001), respectively. This indicates that the antioxidant activities of plant varieties tend to increase when the content of compounds that cause oxidative damage, such as MDA, increases. This is supported by other previous research findings (Yavuz et al., 2023) that reported that stress tends to increase oxidative parameters, e.g., MDA, and trigger antioxidant activities. In addition, the significant positive correlation between phenol and flavonoid antioxidants and proline accumulation (r= 0.64, r=0.68, *p* < 0.001) suggested that plants developed defensive mechanisms to cope with salinity stress through osmotic adjustment and increased antioxidant activities. Our findings revealed that ‘Pawi-2’ had higher phenol and flavonoid contents than the other varieties across all salt concentrations. Hence, it is the most salt-tolerant variety among the studied soybean varieties. This is in agreement with earlier findings reported by Das et al. (2022); Emami et al. (2019); Hossain et al. (2022); Olfa et al. (2018), and Valifard et al. (2014) demonstrating that salt-tolerant plant species have greater antioxidant activity (high phenol and flavonoid contents) than sensitive plant species for detoxifying ROS.

## 5 Conclusion

In this study, we evaluated the effects of salinity across various NaCl labels on the morphological, physiological, and biochemical parameters of soybean plants. Salinity caused a significant morphological, physiological, and biochemical changes in all the studied soybean varieties. This including reductions in plant height, number of leaves per plant, stem thickness, and length and biomass of the shoots and roots as well as an increases in EL, proline, MDA, and total phenol and flavonoid contents. Our results revealed that there was greater variation among varieties in response to salinity stress. A greater reduction in biomass production and other morphological traits was observed in ‘Afigat’. Similarly, the variety developed less osmotic adjustment (low proline content) and antioxidant activity (fewer phenols and flavonoids) and greater membrane damage (high EL) than the other varieties. ‘Pawi-2’ exhibited an increase biomass production, enhance its osmotic adjustment via the accumulation of highly compatible solutes (proline), relatively maintain its membranes (low MDA and EL), and develop antioxidant defensive mechanisms (increased phenol and flavonoid contents). Therefore, ‘Afigat’ and ‘Pawi-2’ were the most salt-sensitive and salt-tolerant varieties, respectively. Further studies in field conditions including anatomical parameters are required to support these findings.

## Data Availability

The original contributions presented in the study are included in the article/Supplementary Material. Further inquiries can be directed to the corresponding authors.

## References

[B1] Abdul Qados A. M. S. (2011). Effect of salt stress on plant growth and metabolism of bean plant Vicia faba (L.). J. Saudi Soc Agric. Sci. 10, 7–15. doi: 10.1016/j.jssas.2010.06.002

[B2] Abeje A. Alemayehu G. Feyisa T. (2021). Nodulation, growth, and yield of soybean (Glycine max L. Merrill) as affected by bio-, organic, and inorganic NPSB fertilizers, and lime in assosa zone, Western Ethiopia. Int. J. Agron. 2021, 1–12. doi: 10.1155/2021/1285809

[B3] Abulfaraj A. A. Jalal R. S. (2021). Use of plant growth-promoting bacteria to enhance salinity stress in soybean (Glycine max L.) plants. Saudi J. Biol. Sci. 28, 3823–3834. doi: 10.1016/j.sjbs.2021.03.053 34220237 PMC8241701

[B4] Adhanom O. G. (2019). Salinity and sodicity hazard characterization in major irrigated areas and irrigation water sources, Northern Ethiopia. Cogent Food Agric. 5, 1–15. doi: 10.1080/23311932.2019.1673110

[B5] Ahmed S. Ahmed S. Roy S. K. Woo S. H. Sonawane K. D. (2019). Effect of salinity on the morphological, physiological and biochemical properties of lettuce (Lactuca sativa L.) in Bangladesh. Open Agric. 4, 361–373. doi: 10.1515/opag-2019-0033

[B6] Aini N. Setiawan A. (2014). Growth and physiological characteristics of soybean genotypes (Glycine max L.) toward salinity stress. AGRIVITA. 36, 201–209. doi: 10.17503/Agrivita-2014-36-3-201-209

[B7] Ajithkumar P. (2017). Morphological and biochemical response to salinity stress on Setaria italica seedlings. J. Appl. Adv. Res. 2, 235–248. doi: 10.21839/jaar.2017.v2i4.96

[B8] Alebel M. G. Urge M. Assefa G. Worku B. Abebe A. (2019). The effect of using either soybean or groundnut straw as part of basal diet on body weight gain, and carcass characteristics of Gumuz Sheep. Int. J. Livest. Prod. 10, 70–76. doi: 10.5897/IJLP2018.0549

[B9] Alharby H. F. Nahar K. Al-Zahrani H. S. Hakeem K. R. Hasanuzzaman M. (2021). Enhancing salt tolerance in soybean by exogenous boron: Intrinsic study of the ascorbate-glutathione and glyoxalase pathways. Plants 10, 2085–2097. doi: 10.3390/plants10102085 34685894 PMC8537241

[B10] Amirjani M. R. (2010). Effect of salinity stress on growth, mineral composition, proline content, antioxidant enzymes of soybean. Am. J. Plant Physiol. 5, 350–360. doi: 10.3923/ajpp.2010.350.360

[B11] Anwar-ul-haq M. Akhtar J. Basra S. M. A. (2016). Interactive effect of salinity and potassium on growth, biochemical parameters, protein and oil quality of soybean genotypes. Pak. J. Agric. Sci. 53, 69–78. doi: 10.21162/PAKJAS/16.4755

[B12] Anwar-ul-haq M. Aziz T. Aziz O. (2020). Potassium induces carbohydrates accumulation by enhancing morpho-physiological and biochemical attributes in soybean under salinity. Arch. Agron. Soil Sci. 00, 1–14. doi: 10.1080/03650340.2020.1769075

[B13] Aredehey G. Libsekal H. Brhane M. Welde K. Giday A. (2018). Top-soil salinity mapping using geostatistical approach in the agricultural landscape of Timuga irrigation scheme, South Tigray, Ethiopia. Cogent Food Agric. 4, 1–13. doi: 10.1080/23311932.2018.1514959

[B14] Argaw A. (2014). Response of soybean to inoculation with bradyrhizobium spp. in saline soils of shinille plains, Eastern Ethiopia. East Afr. J. Sci. 8, 79–90.

[B15] Arnon D. A. L. I. (1949). Plant physiology. Plant Physiol. 24, 1–15. doi: 10.2307/4118807 16654194 PMC437905

[B16] Ashraf A. M. Ali Q. (2010). Response of two genetically diverse wheat cultivars to salt stress at different growth stages: leaf lipid peroxidation and phenolic contents. Pak. J. Bot. 42, 559–565.

[B17] Ashraf M. Harris P. J. C. (2013). Photosynthesis under stressful environments: An overview. Photosynthetica 51, 163–190. doi: 10.1007/s11099-013-0021-6

[B18] Awoke B. Alem M. B. (2021). Effects of saline water and irrigation interval on soybean (Glycine max) yield. J. Civ. Environ. Eng. 11, 1–7.

[B19] Bates L. S. And R. P. W. Teare I. D. (1973). Rapid determination of free proline for water-stress studies. Plant Soil. 39, 205–207.

[B20] Carloni E. Quiroga M. Tommasino E. Griffa S. Grunberg K. Ribotta A.. (2012). Grass and Forage Science Malondialdehyde content as a potential biochemical indicator of tolerant Cenchrus ciliaris L. genotypes under heat stress treatment. Grass Forage Sci. 67, 456–459. doi: 10.1111/j.1365-2494.2012.00851.x

[B21] Chang C. Yang M. Wen H. Chern J. (2002). Estimation of total flavonoid content in propolis by two complementary colorimetric methods. J. Food Drug Anal. 10, 178–182. doi: 10.38212/2224-6614.2748

[B22] Chutipaijit S. Cha-Um S. Sompornpailin K. (2009). Differential accumulations of proline and flavonoids in indica rice varieties against salinity. Pakistan J. Bot. 41, 2497–2506.

[B23] Das A. K. Anik T. R. Rahman M. Keya S. S. (2022). Ethanol treatment enhances physiological and biochemical responses to mitigate saline toxicity in soybean. Plants Artic. 11, 1–18. doi: 10.3390/plants11030272 PMC8838166 35161252

[B24] Dayem A. A. Hossain M. K. Lee S.B. Kim K. Saha S. K. Yang G. M.. (2017). The role of reactive oxygen species (ROS) in the biological activities of metallic nanoparticles. Int. J. Mol. Sci. 18, 1–21. doi: 10.3390/ijms18010120 PMC5297754 28075405

[B25] Dichala O. Giannakoula A. E. (2022). Effect of salinity on physiological and biochemical parameters of leaves in three pomegranate (Punica granatum L.) cultivars. Appl. Sci. Artic. 12, 1–13. doi: 10.3390/app12178675

[B26] Dionisio-Sese M. L. Tobita S. (1998). Antioxidant responses of rice seedlings to salinity stress. Plant Sci. 135, 1–9. doi: 10.1016/S0168-9452(98)00025-9

[B27] Ejeta M. (2020). Effect of Priming on Seed Quality of Soybean [Glycine max (L.) Merrill] Varieties at Assosa, Western Ethiopia. Sci. Res. 8, 59–72. doi: 10.11648/j.sr.20200803.11

[B28] El kholy R. Sayed A. I. EL-Shaer H. F. Hanafy M. S. (2021). Impact of sea salt stress on growth and some physiological attributes of two soybean (Glycine Max L.) cultivars. Al-Azhar J. Agric. Res. 6, 1559–1571. doi: 10.21608/ajar.2021.245619

[B29] Emami Z. Hashemi M. Dacosta M. Craker L. Maggi F. (2019). Industrial Crops & Products E ff ect of salinity stress on the physiological characteristics, phenolic compounds and antioxidant activity of Thymus vulgaris L. and Thymus daenensis Celak. Ind. Crop Prod. 135, 311–320. doi: 10.1016/j.indcrop.2019.04.055

[B30] Ertump M. Dobo B. Mikru A. (2019). Effect of Arbuscular Mycorrhizal Fungi Inoculation on Growth and Mineral Nutrition of Soybean (G lycine max) Grown Under Different Salinity Levels. Inernational J. Agric. Innov. Res. 8, 171–187.

[B31] Fatema M. K. Abdullah M. Mamun A. Sarker U. Hossain M. S. Abdul M.. (2023). Assessing morpho-physiological and biochemical markers of soybean for drought tolerance potential. Sustain. Artic. 15, 1–19. doi: 10.3390/su15021427

[B32] Foliar L. Oxide N. Sardar H. Khalid Z. Ahsan M. Naz S.. (2023). Enhancement of Salinity Stress Tolerance in Lettuce (Lactuca sativa L.) via Foliar Application of Nitric Oxide. Plants Artic. 12, 1–24. doi: 10.3390/plants12051115 PMC10005404 36903975

[B33] Gomes M. A. da C. Pestana I. A. Santa-Catarina C. Hauser-Davis R. A. Suzuki M. S. (2017). Salinity effects on photosynthetic pigments, proline, biomass and nitric oxide in Salvinia auriculata Aubl. Acta Limnol. Bras. 29, 1–13. doi: 10.1590/s2179-975x4716

[B34] Habibi N. Sediqui N. Terada N. Sanada A. Koshio K. (2021). Effects of salinity on growth, physiological and biochemical responses of tomato. J. Int. Soc Southeast Asian Agric. Sci. 27, 14–18.

[B35] Hanifah N. Purwestri Y. A. (2021). The effect of naCl salinity stress to phenolic compound, total flavonoid and antioxidant activity of pegagan (Centella asiatica (L.) urban) leaves. Bio Web Conf. 41, 06004–06009. doi: 10.1051/bioconf/20214106004

[B36] Hasanuzzaman M. Nahar K. Rahman A. Mahmud J. A. Hossain M. S. Fujita M. (2016). Soybean production and environmental stresses (Elsevier Inc). doi: 10.1016/B978-0-12-801535-3.00004-8

[B37] Hasanuzzaman M. Raihan M. R. H. Masud A. A. C. Rahman K. Nowroz F. Rahman M.. (2021). Regulation of reactive oxygen species and antioxidant defense in plants under salinity. Int. J. Mol. Sci. 22, 9326–9355. doi: 10.3390/ijms22179326 34502233 PMC8430727

[B38] Hasanuzzaman M. Raihan M. R. H. Nowroz F. Fujita M. (2022). Insight into the Mechanism of Salt-Induced Oxidative Stress Tolerance in Soybean by the Application of Bacillus subtilis: Coordinated Actions of Osmoregulation, Ion Homeostasis, Antioxidant Defense, and Methylglyoxal Detoxification. Antioxidants 11, 1856–1877. doi: 10.3390/antiox11101856 36290578 PMC9598349

[B39] Heath R. L. Packer L. (1968). Photoperoxidation in isolated chloroplasts. Arch. Biochem. Biophys. 125, 189–198. doi: 10.1016/0003-9861(68)90654-1 5655425

[B40] Heidari M. (2011). Effects of salinity stress on growth, chlorophyll content and osmotic components of two basil (Ocimum basilicum L.) genotypes. Afr. J. Biotechnol. 11, 379–384. doi: 10.5897/ajb11.2572

[B41] Heidari M. (2012). Effects of salinity stress on growth, chlorophyll content and osmotic components of two basil (Ocimum basilicum L.) genotypes. Afr. J. Biotechnol. 11, 379–384. doi: 10.5897/AJB11.2572

[B42] Hniličková H. Hnilička F. Orsák M. Hejnák V. (2019). Effect of salt stress on growth, electrolyte leakage, Na + and k + content in selected plant species. Plant Soil Environ. 65, 90–96. doi: 10.17221/620/2018-PSE

[B43] Hossain N. Sarker U. Raihan S. Al-huqail A. A. Siddiqui M. H. (2022). Influence of Salinity Stress on Color Parameters, Leaf Pigmentation, Polyphenol and Flavonoid Contents, an d Antioxidant Activity of Amaranthus lividus Leafy Vegetables. Mol. Artic. 27, 1–19. doi: 10.3390/molecules27061821 PMC8955103 35335185

[B44] Hunde Desissa D. (2019). Soybean research and development in Ethiopia. Acta Sci. Agric. 3, 192–194. doi: 10.31080/asag.2019.03.0668

[B45] Irin I. J. Hasanuzzaman M. (2024). Organic amendments: enhancing plant tolerance to salinity and metal stress for improved agricultural productivity. Stresses 4, 185–209. doi: 10.3390/stresses4010011

[B46] Ji X. Tang J. Zhang J. (2022). Effects of salt stress on the morphology, growth and physiological parameters of Juglans microcarpa L. seedlings. Plants Artic. 11, 1–21. doi: 10.3390/plants11182381 PMC9506368 36145780

[B47] Joshi A. Rajput V. D. Verma K. K. Minkina T. Ghazaryan K. Arora J. (2023). Potential of Suaeda nudiflora and Suaeda fruticosa to Adapt to High Salinity Conditions. Hortic. Artic. 9, 1–18. doi: 10.3390/horticulturae9010074

[B48] Kafi M. Nabati J. Masoumi A. L. I. Mehrgerdi M. Z. (2011). EFFECT OF SALINITY AND SILICON APPLICATION ON OXIDATIVE DAMAGE OF SORGHUM [SORGHUM BICOLOR (L.) MOENCH. Pak. J. Bot. 43, 2457–2462.

[B49] Kafi M. Rahimi Z. (2011). Effect of salinity and silicon on root characteristics, growth, water status, proline content and ion accumulation of purslane (Portulaca oleracea L.). Soil Sci. Plant Nutr. 57, 341–347. doi: 10.1080/00380768.2011.567398

[B50] Kao C. H. (2017). Mechanisms of salt tolerance in rice plants: reactive oxygen species scavenging-systems. J. Taiwan Agric. Res. 66, 1–8. doi: 10.6156/JTAR/2017.06601.01

[B51] Ke W. Hou H. (2021). Effect of salt stress on growth, physiological parameters, and ionic concentration of water dropwort (Oenanthe javanica) cultivars. Front. Plant Sci. 12. doi: 10.3389/fpls.2021.660409 PMC8256277 34234795

[B52] Kesawat M. S. Satheesh N. Kherawat B. S. Kumar A. Kim H. U. Chung S. M.. (2023). Regulation of reactive oxygen species during salt stress in plants and their crosstalk with other signaling molecules—Current perspectives and future directions. Plants 12, 864–897. doi: 10.3390/plants12040864 36840211 PMC9964777

[B53] Khan F. Siddiqi T. O. Mahmooduzzafar Ahmad A. (2009). Morphological changes and antioxidant defence systems in soybean genotypes as affected by salt stress. J. Plant Interact. 4, 295–306. doi: 10.1080/17429140903082635

[B54] Khan M. A. Sahile A. A. Jan R. Asaf S. Hamayun M. Imran M.. (2021). Halotolerant bacteria mitigate the effects of salinity stress on soybean growth by regulating secondary metabolites and molecular responses. BMC Plant Biol. 21, 1–15. doi: 10.1186/s12870-021-02937-3 33845762 PMC8040224

[B55] Kiani R. Arzani A. Mirmohammady Maibody S. A. M. (2021). Polyphenols, flavonoids, and antioxidant activity involved in salt tolerance in wheat, aegilops cylindrica and their amphidiploids. Front. Plant Sci. 12. doi: 10.3389/fpls.2021.646221 PMC8027307 33841475

[B56] Liao R. Xu Y. (2021). Effects of salt stress on the physiological characteristics of Solanum photeinocarpum. E3S Web Conf. 233, 1–4. doi: 10.1051/e3sconf/202123301108

[B57] Liu T. van Staden J. (2000). Selection and characterization of sodium chloride-tolerant callus of Glycine max (L.) Merr cv. Acme. Plant Growth Regul. 31, 195–207. doi: 10.1023/A:1006391400927

[B58] Mane A. V. Karadge B. A. Samant J. S. (2010). Salinity induced changes in photosynthetic pigments and. J. Chem. Pharm. 2, 338–347.

[B59] Mankar G. D. Wayase U. R. Shelke D. B. Nikam T. D. Barmukh R. B. (2021). Morphological, physiological, and biochemical responses to NaCl-induced salt stress in mungbean (Vigna radiata L.) varieties. Not. Sci. Biol. 13, 1–17. doi: 10.15835/nsb13210936

[B60] Mohabbati F. (2005). Effects of salinity on syntethic wheat genotypes. Czech J. Genet. Plant Breed. 41, 268–272. doi: 10.17221/6189-cjgpb

[B61] Molla H. S. Ayele Z. A. Zeleke M. A. (2024). Trends, opportunities, and challenges of the Ethiopian soybean export market in the past two decades, (2004-2022). Adv. Agric. 2024, 1–8. doi: 10.1155/2024/9979892

[B62] Momeni M. M. Kalantar M. Dehghani-Zahedani M. (2021). Physiological, biochemical and molecular responses of durum wheat under salt stress. Plant Genet. Resour. Characterisation Util. 19, 93–103. doi: 10.1017/S1479262120000416

[B63] Mussema R. Diro S. Erko B. Teshale D. Dibaba R. Abush Tesfaye A.. (2022). Soybean value chain analysis in Ethiopia: A qualitative study research report number: 134. Available online at: http://www.eiar.gov.et (Accessed date 17 December 2021).

[B64] Mwendwa S. (2020). The Mwendwa Protocol : A modi cation of the Bouyoucos method of soil texture analysis. Res. Sq., 1–20.

[B65] Nguyen M. K. Yang C. M. Shih T. H. Lin S. H. Pham G. T. Nguyen H. C. (2021). Chlorophyll biosynthesis and transcriptome profiles of chlorophyll b-deficient type 2b rice (Oryza sativa L.). Not. Bot. Horti Agrobot. Cluj-Napoca 49, 1–16. doi: 10.15835/nbha49312380

[B66] Olfa B. Maha Z. Nada B. Zeineb O. A. (2018). Effects of NaCl on plant growth and antioxidant activities in fenugreek (Trigonella foenum graecum L.). Biosci. J. Uberlândia 34, 683–696.

[B67] Oprica L. Marius Ş. (2014). Evaluation of morphological and biochemical parameters of soybean seedlings induced by saline stress. Rom. Biotechnol. Lett. 19, 9615–9624.

[B68] Pacheco-Sangerman F. Gómez-Merino F. C. Peralta-Sánchez M. G. Trejo-Téllez L. I. (2024). Sulfated nutrition modifies nutrient content and photosynthetic pigment concentration in cabbage under salt stress. Plants 13, 1337–1350. doi: 10.3390/plants13101337 PMC11124958 38794408

[B69] Pradeep H. K. Balasangameshwara J. Rajan K. Madhu M. Archana B. K. (2021). Automation of USDA triangle soil texture classification using finite state machine : A novel conceptual modeling approach. Stat. Appl. 19, 255–266.

[B70] Qureshi A. S. Ertebo T. Mehansiwala M. (2018). Prospects of alternative copping systems for salt- affected soils in Ethiopia. J. Soil Sci. Environ. Manage. Rev. 9, 98–107. doi: 10.5897/JSSEM2018.0686

[B71] Rahman M. S. Malek M. A. Emon R. M. Hannan A. Sagor G. H. M. (2020). morphological and molecular characterization of soybean (Glycine max L.) genotypes under salt stress. Ann. Bangladesh Agric. 24, 33–46. doi: 10.3329/aba.v24i2.55782

[B72] Rahmawati N. Damanik R. I. M. (2018). Effect of foliar application of α-tocopherol on vegetative growth and some biochemical constituents of two soybean genotypes under salt stress. IOP Publishing Ltd. doi: 10.1088/1755-1315/122/1/012049

[B73] Rasel M. Tahjib-Ul-Arif M. Hossain M. A. Hassan L. Farzana S. Brestic M. (2021). Screening of salt-tolerant rice landraces by seedling stage phenotyping and dissecting biochemical determinants of tolerance mechanism. J. Plant Growth Regul. 40, 1853–1868. doi: 10.1007/s00344-020-10235-9

[B74] Ricardo B. Maria E. Guedes S. Alves L. Pereira R. M. Batista B. L.. (2023). How different na + Concentrations affect anatomical, nutritional physiological, biochemical, and morphological aspects in soybean plants : A multidisciplinary and comparative approach. Agron. Artic. 13, 1–21. doi: 10.3390/agronomy13010232

[B75] Rout N. P. Tripathi S. B. Shaw B. P. (1997). Effect of salinity on chlorophyll and proline contents in three aquatic macrophytes. Biol. Plant 40, 453–458. doi: 10.1023/A:1001186502386

[B76] Saberi A. R. Siti Aishah H. Halim R. A. Zaharah A. R. (2011). Morphological responses of forage sorghums to salinity and irrigation frequency. Afr. J. Biotechnol. 10, 9647–9656. doi: 10.5897/AJB11.778

[B77] Sabra A. Daayf F. Renault S. (2012). Differential physiological and biochemical responses of three EChinacea species to salinity stress. Sci. Hortic. (Amsterdam). 135, 23–31. doi: 10.1016/j.scienta.2011.11.024

[B78] Saxena A. Purty R. S. (2018). Evaluating morphological, physiological and biochemical responses of three amphiploid brassica species to salinity stress. Indian J. Biol. 4, 10–18. doi: 10.21088/ijb.2394.1391.4117.2

[B79] Sharma P. Jha A. B. Dubey R. S. Pessarakli M. (2012). Reactive oxygen species, oxidative damage, and antioxidative defense mechanism in plants under stressful conditions. J. Bot. 2012, 1–26. doi: 10.1155/2012/217037

[B80] Siddiki S. A. Wagh S. G. Sul R. S. Pawar K. R. Harke S. N. (2020). Comparative Studies among Different Genotypes of Soybean (Glycine max L.) against Salinity Stress. Curr. J. Appl. Sci. Technol. 39, 91–100. doi: 10.9734/cjast/2020/v39i630563

[B81] Singh D. (2022). Juggling with reactive oxygen species and antioxidant defense system – A coping mechanism under salt stress. Plant Stress 5, 100093–100105. doi: 10.1016/j.stress.2022.100093

[B82] Sonobe R. Yamashita H. Mihara H. Morita A. Ikka T. (2020). Estimation of leaf chlorophyll a, b and carotenoid contents and their ratios using hyperspectral reflectance. Remote Sens. 12, 1–19. doi: 10.3390/rs12193265

[B83] Sonone M. Mane A. Sawardekar S. (2023). Consequences of salt stress on chlorophyll pigments of rice genotypes. Pharma Innov. J. 12, 3272–3275.

[B84] Tessema N. Yadeta D. Kebede A. Ayele G. T. (2023). Soil and irrigation water salinity, and its consequences for agriculture in Ethiopia : A systematic review. Agric. Rev. 13, 1–19. doi: 10.3390/agriculture13010109

[B85] Trina F. A. Ahmed R. Ruhi R. A. Joy M. I. H. Maliha M. B. J. (2021). Morphological performances of BINA soybean 6 (Glycine max) at several salinity stress concentrations in coastal region of Bangladesh. J. Biosci. Agric. Res. 27, 2287–2295. doi: 10.18801/jbar.270221.278

[B86] Tuwair K. Al S. Farag K. M. (2015). The use of electrolyte leakage procedure in assessing heat and salt tolerance of Ruzaiz date palm (Phoenix dactylifera L.) cultivar regenerated by tissue culture and offshoots and treatments to alleviate the stressful injury. J. Hortic. For. 7, 104–111. doi: 10.5897/JHF2014.0378

[B87] Ullah N. Basit A. Ahmad I. Ullah I. Shah S. T. Mohamed H. I. (2020). Mitigation the adverse effect of salinity stress on the performance of the tomato crop by exogenous application of chitosan. Bull. Natl. Res. Cent. 44, 1–11. doi: 10.1186/s42269-020-00435-4

[B88] Valifard M. Mohsenzadeh S. Kholdebarin B. Rowshan V. (2014). Effects of salt stress on volatile compounds, total phenolic content and antioxidant activities of Salvia mirzayanii. South Afr. J. Bot. 93, 92–97. doi: 10.1016/j.sajb.2014.04.002

[B89] Wasif M. Shafiqullah A. Ahmad A. Samadi F. (2023). Interpretation of morpho − physiological and biochemical responses of winter wheat under different sodium chloride concentrations. J. Crop Sci. Biotechnol. 26, 563–571. doi: 10.1007/s12892-023-00200-9

[B90] Wu G. Zhou Z. Chen P. Tang X. Shao H. Wang H. (2014). Comparative ecophysiological study of salt stress for wild and cultivated soybean species from the yellow river delta, China. Sci. World J. 2014, 1–13. doi: 10.1155/2014/651745 PMC4066866 24999494

[B91] Xiao-shan W. Jian-guo H. A. N. (2009). Changes of proline content, activity, and active isoforms of antioxidative enzymes in two alfalfa cultivars under salt stress. Agric. Sci. China 8, 431–440. doi: 10.1016/S1671-2927(08)60229-1

[B92] Yavuz D. Seymen M. Kal Ü. Atakul Z. Tanrıverdi Ö.B. Türkmen Ö.. (2023). Agronomic and physio-biochemical responses of lettuce to exogenous sodium nitroprusside (SNP) applied under different irrigation regimes. Agric. Water Manage. 277, 1–11. doi: 10.1016/j.agwat.2022.108127

[B93] Zhou Y. Tang N. Huang L. Zhao Y. Tang X. Wang K. (2018). Effects of salt stress on plant growth, antioxidant capacity, glandular trichome density, and volatile exudates of schizonepeta tenuifolia briq. Int. J. Mol. Sci. Artic. 19, 252–266. doi: 10.3390/ijms19010252 PMC5796199 29342961

