# Supplementary material for: Morphological, physiological, and biochemical responses of three different soybean (Glycine max L.) varieties under salinity stress conditions
Source: Front Plant Sci. 2024 Sep 9;15:1440445. doi: 10.3389/fpls.2024.1440445 (PMC11443463; doi:10.3389/fpls.2024.1440445)
Supplement: Supplementary file 1 [file DataSheet1.docx]

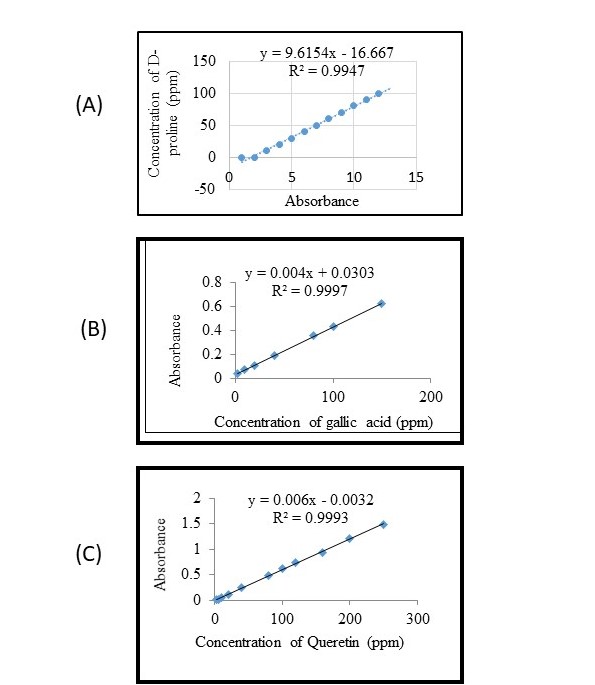


Figure. Standard curves for the determination leave proline content (A), total phenol content (b) and total flavonoid content (C) of three soybean plant varieties.
